# Anatomical landmarks are more accurate in identifying the ideal femoral insertion for modified Larson reconstruction of posterolateral corner than radiological landmarks

**DOI:** 10.1002/jeo2.70282

**Published:** 2025-05-19

**Authors:** Christian Coppola, Maximilian Sigloch, Romed Hörmann, Werner Schmoelz, Raul Mayr

**Affiliations:** ^1^ Department of Orthopedics and Trauma Surgery Medical University of Innsbruck Innsbruck Austria; ^2^ Department of Anatomy Medical University of Innsbruck Innsbruck Austria

**Keywords:** biomechanics, isometric point, knee dislocation, modified Larson technique, posterolateral reconstruction

## Abstract

**Purpose:**

In surgery for posterolateral knee instabilities, the modified Larson technique (MLT) is a fibular tunnel–based reconstruction technique with a single femoral tunnel aiming for an isometric graft insertion point (IGIP). The IGIP can be located intraoperatively using an anatomically referenced method (ARM) or a radiological method (RM). The purpose of this experimental study was to compare the ARM with the RM in terms of isometric behaviour and to report the location of the ARM and RM in relation to the lateral epicondyle (LE).

**Methods:**

Flexion/extension movement of eight fresh‐frozen human knee joints was simulated in a custom‐made knee test bench. A fibular tunnel was created as described in the MLT, and a suture was shuttled from the IGIP through the tunnel and connected to a displacement transducer. The isometry of the IGIP of the ARM and RM was evaluated on the basis of suture displacement during flexion/extension motion. The position of the determined IGIP relative to the centre of the LE was measured on true lateral X‐rays.

**Results:**

Comparison of the isometry behaviour of the two techniques showed that RM resulted in a displacement of 10.46 ± 3.69 mm, whereas the ARM showed a of 6.09 ± 2.11 mm during flexion/extension motion (*p* = 0.017). The median location of the ARM and RM was 6.5 mm (IQR 8.375 mm), 5.45 mm (IQR 3.5 mm) distal and 3.95 mm (IQR 6.9 mm), 4.55 mm (IQR 5.75 mm) anterior to the centre of the LE, respectively.

**Conclusions:**

In the present in vitro experiment, the ARM was capable of determining the femoral IGIP more accurately than the radiological method. For clinical practice, it is recommended to start approximately 6.5 mm distal and 3.95 mm anterior to the centre of the LE in order to determine the IGIP when performing MLT.

**Abbreviations**
ARM: anatomically referenced method
IGIP: isometric graft insertion point
IQR: interquartile range
LCL: lateral collateral ligament
LE: lateral epicondyle
LVDT: linear variable displacement transducer
MLT: modified Larson technique
PT: popliteus tendon
RM: radiological method
SD: standard deviation

## INTRODUCTION

In surgery for posterolateral knee instabilities, the modified Larson technique (MLT) is a fibular tunnel–based reconstruction technique with a single femoral tunnel aiming for an isometric graft insertion point (IGIP) [11, 24]. The femoral IGIP can be located intraoperatively on the basis of an anatomically referenced method (ARM) or using a radiological method (RM) [12, 18]. However, determining the IGIP remains challenging with the MLT [3, 17]—partly because an ‘isometric point’ does not really exist, as knee biomechanics depend on a complex roll–glide motion mechanism [3, 7, 18]. Especially in treatment of knee dislocations and multiligament knee injuries, determination of the IGIP continues to be demanding as soft tissue landscape may be distorted [3, 17]. Intraoperative identification of the IGIP through an ARM uses the lateral femoral epicondyle (LE) as an anatomic reference and starting point, before moving in all cardinal directions in order to identify the point that has the most isometric behaviour for the MLT graft [11, 14, 24]. As an alternative, the RM can be used to identify the rotational axis of the knee joint and orientation for lateral knee reconstructions, as previously described by Stannard and Schmidt [19]. The RM involves identifying the intersection of the posterior cortex of the femur and the Blumensaat line on a strictly lateral radiograph of the femur. Although there have been studies reporting on the accuracy of the RM [18], it is still unclear whether a radiologically determined lateral IGIP is valid for the MLT [8, 12, 18]. It is also not been sufficiently investigated which method is more accurate in determining the IGIP. Furthermore, identification of the IGIP may represent an important, however often overlooked point of single femoral tunnel reconstruction techniques. For this reason, to support intraoperative decision making, advanced knowledge about the anatomical location of the IGIP in relation to the LE was the focus of the present study.

The first purpose of this experimental study was to compare the isometric behaviour of insertion points determined using the ARM with those determined by the RM, by measuring suture displacements. The second aim was to report the radiological location of the ARM and RM relative to the LE.

## MATERIALS AND METHODS

### Specimen preparation

Eight fresh‐frozen human knee joints from donors with a median age of 74.9 ± 5.4 years (three men, five women) were obtained from the local anatomical institute and used for this biomechanical in vitro study [2]. Before testing, computed tomography was carried out in order to exclude severe knee arthritis (Kellgren–Lawrence ≥ 3), to ensure a focus on mild to moderate cases and avoid advanced joint damage that could distort results [6]. After skin and subcutaneous tissue had been removed, the specimens were inspected for the integrity of the ligaments and joint capsule. Once all the specimens had been osteotomized to the same length, the fibula was fixed to the tibia using two screws, in order to secure it against rotation [1]. Using a custom‐made rig, embedding of the tibia and fibula was performed using polymethylmethacrylate cement (Technovit 3040, Kulzer GmbH, Wehrheim, Germany). The knee was then placed in a custom‐made test bench for testing [4]. A fibular tunnel was created as described in the MLT: [11] After dissection of the ventral peroneal muscle at the transition from the fibula shaft to the fibula head, a k‐wire was drilled in an anterolateral to posteromedial direction to the contralateral cortex. After checking of the k‐wire position under an image intensifier, it was over‐drilled with a 5‐mm headspace drill.

### Radiological and anatomically referenced method

**Figure 1:**
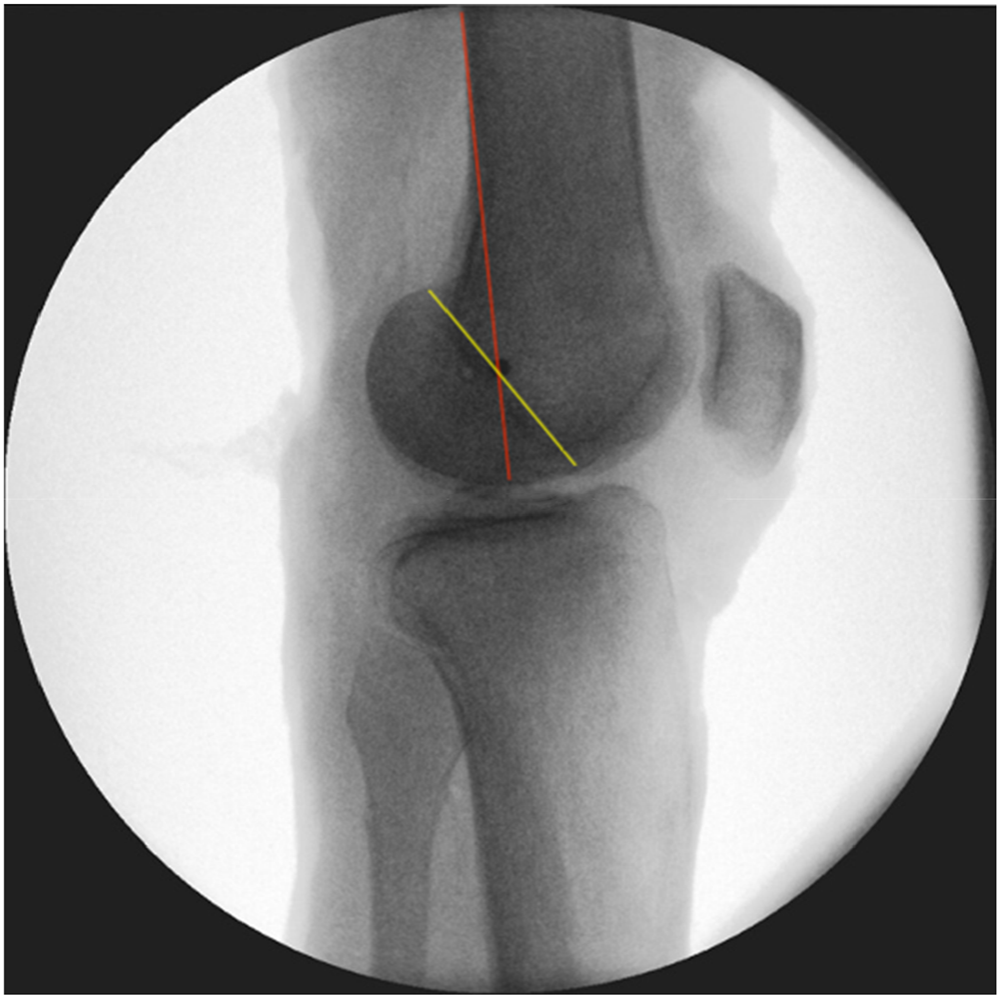
The radiological method (RM) [19]. In a straight lateral x‐ray, a k‐wire is placed at the intersection between a line that extends the posterior cortex of the femur (red line) and the Blumensaat line (yellow line). If it is aligned parallel relative to the knee joint line in an anteroposterior view, it appears as a single dot.

The RM was applied as described by Stannard et al. [18, 19], with the isometric point being determined on a strictly lateral radiograph of the femur by the intersection of a line drawn from the posterior cortex of the femur and the Blumensaat line (Figure 1). Once this point was identified, the K‐wire was placed parallel to the fluoroscope until it appeared as a single dot in the lateral view and parallel to the knee joint in the anteroposterior view.

In the ARM, a custom‐made eyelet k‐wire was drilled 7.5 mm anterior to the centre of the LE toward the medial side [11]. Following this step, four further drilling procedures were carried out, each in a different cardinal direction, in a standardised distance of 7.5 mm. For each of the five drilling procedures, the isometry was experimentally tested and the most isometric trial was taken.

### Test setup and data analysis

**Figure 2:**
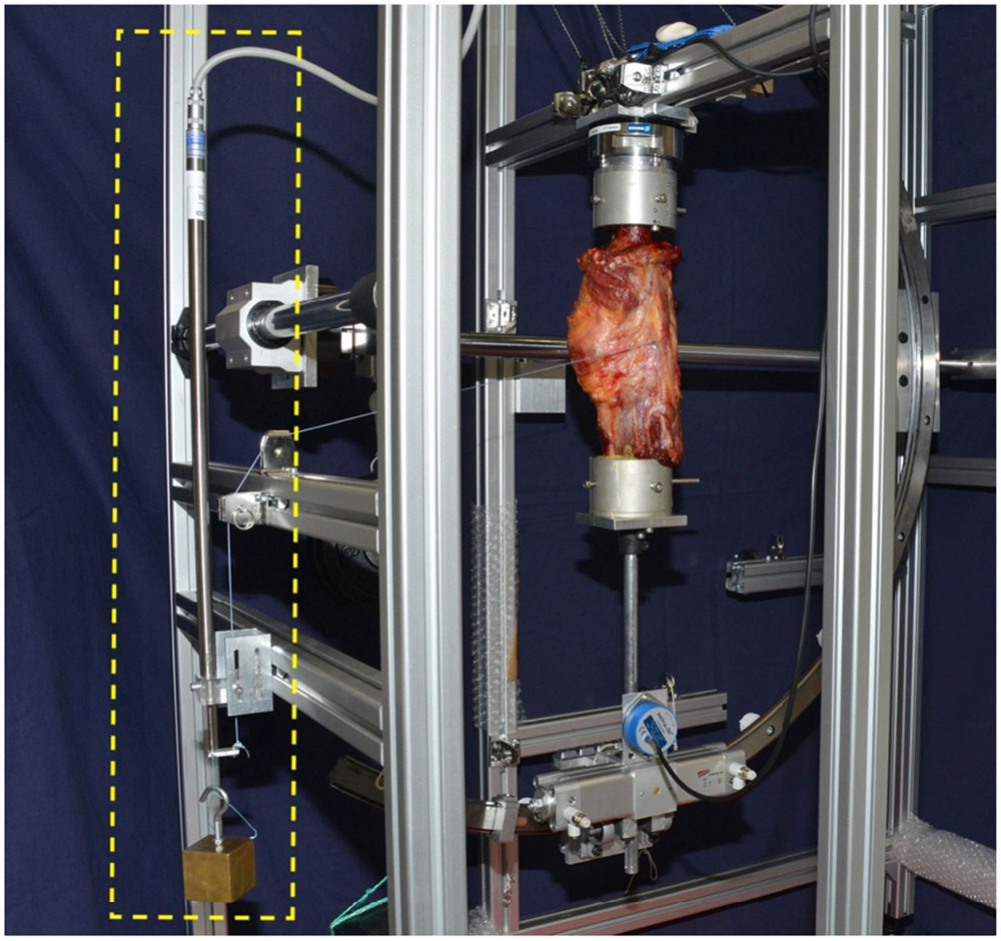
In a custom‐made test bench, suture displacement is measured by a linear variable displacement transducer (LVDT; yellow box) while the specimen is flexed from a neutral joint position (0°) to 120° of flexion and back.

**Figure 3:**
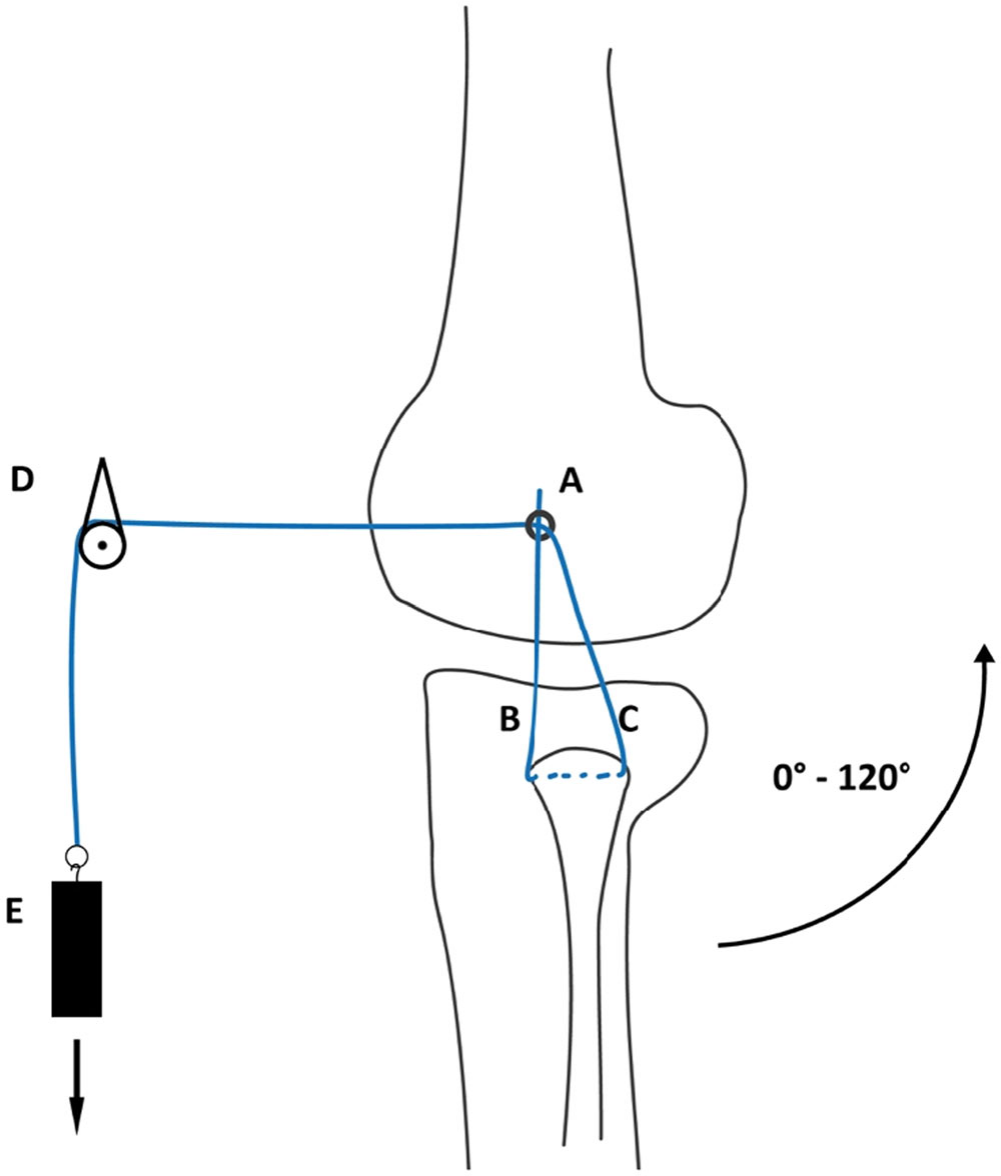
Schematic depiction of the suture path, including deflections. A, a custom‐made eyelet k‐wire is placed at the chosen point with the attached suture; B, the descending limb of the suture before it passes through the fibular tunnel; C, the ascending limb of the suture, which is shuttled below the biceps tendon and passed anteriorly through the eyelet; D, deflection of the suture; E, suture anchorage on a weight (500 g).

To simulate the displacement of a reconstructed ligament inserted into the chosen IGIP, a nonabsorbable surgical suture (Fiberwire No. 2; Arthrex Inc., Naples, Florida) was used as a graft surrogate and connected to a custom‐designed length measuring system (Figures 2 and 3) with a linear variable displacement transducer (LVDT) (Messotron GmbH, Seeheim‐Jugenheim, Germany). The suture was pulled through the fibular channel to simulate the reconstructed ligament. The anterior, ascending limb was attached to an eyelet, which was positioned on the femoral insertion point. The posterior limb was shuttled below the biceps tendon and passed anteriorly through the same eyelet and finally attached to a weight (500 g) over a pulley. Knee motion from 0° to 120°of flexion and vice versa was performed five times in order to investigate the whole range of motion required during the most functional activities [5], while the LCL undergoes length changes [10, 23]. To quantify the suture displacement, the differences between the maximum and minimum recorded values with the LVDT during the last three of the five motion cycles were captured. A measuring amplifier (PICAS measuring amplifier; Peekel Instruments GmbH, Bochum, Germany) in combination with a customised software routine (LabVIEW 11.0; National Instruments, Austin, Texas, USA) was used for data acquisition. LVDT readings were verified prior to testing each specimen using a mechanical micrometre screw gauge and care was taken that the expected measurement range was in the linear region of the LVDT. Before each length measurement, the reading of the LVDT was zeroed in the software using a built‐in function. For final evaluation, the data were exported to a spreadsheet programme (Excel 2016; Microsoft Corporation, Redmond, Washington, USA).

### Location analysis

**Figure 4:**
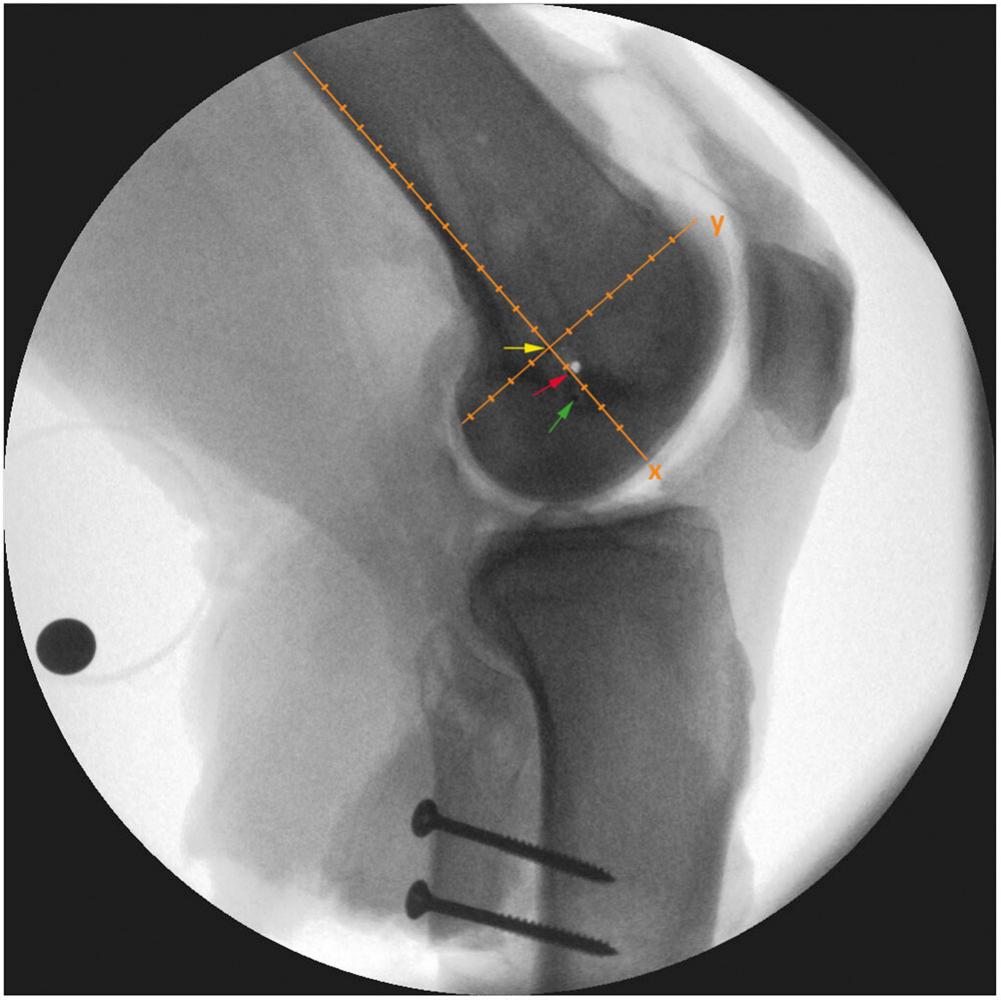
A strictly lateral X‐ray of the femur, showing the custom‐made markers placed on the lateral condyle of the knee joint (three black dots). The markers are visualised using coloured arrows. The proximal marker represents the lateral epicondyle (LE, yellow), the intermediate marker represents the radiological method (RM, red) and the distal one represents the anatomically referenced method (ARM, green). The xy‐coordinate system (orange) and the additional positioning of a calibration sphere allow measurement of real distances from the centre of the lateral epicondyle.

The positional relationships of the IGIP determined using the ARM and RM to the centre of the lateral epicondyle were evaluated. For this purpose, custom‐made markers were placed at each site determined by the ARM and the RM, respectively. A strictly lateral X‐ray of each specimen was taken (Figure 4). Additional positioning of a calibration sphere allowed measurement of real distances, ensuring consistency of the scaling factor between images. Distances in millimetres were measured using an xy‐coordinate system. The alignment of the xy‐coordinate system was defined by a line parallel to the posterior femoral cortex running through the centre of LE. This line was defined as the x‐axis, with a perpendicular y‐axis at the centre of the LE of the femur (Figure 4).

### Statistical analysis

Data for suture displacement of the simulated graft are reported in millimetres (mm) with mean and standard deviation (SD). A nonnormal distribution was assumed, due to the limited number of specimens (*n* = 8). A Wilcoxon signed rank test was used to assess statistical relevance between linked samples. The significance level was set at *p* = 0.05. Descriptive statistics was used to report the position of the ARM and RM relative to the LE. Due to the small sample size (*n* = 8), a nonnormal distribution was assumed, and the results are reported with the median and interquartile range (IQR). Statistical analysis was performed using IBM SPSS Statistics for Windows, version 27.0 (SPSS, IBM Corporation, Armonk, New York, USA).

## RESULTS

### Suture displacement (RM vs. ARM)

**Figure 5:**
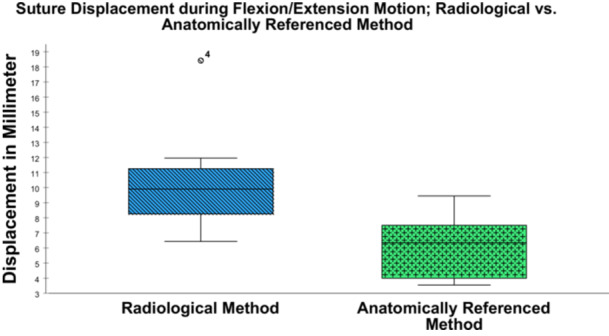
Suture displacement in millimetres (mm) for the radiological method (RM) and anatomically referenced method (ARM) for determining the isometric graft insertion point (IGIP) in the knee joint. The box plots show the median, 25–75th interquartile range, and distributions.

Overall, the ARM showed a mean displacement of 6.09 ± 2.11 mm (95% ‐ CI (4.32–7.86)), whereas the RM resulted in a mean displacement of 10.46 ± 3.69 mm (95% ‐ CI (7.38–13.54)) during flexion/extension motion (*p* = 0.017). During flexion/extension motion, the ARM showed a minimum and maximum displacement of 3.55 mm and 9.45 mm, respectively. In comparison, the RM showed a minimum displacement of 6.44 mm and a maximum displacement of 18.42 mm. The displacement shown by the RM was less than that with the ARM in only one specimen (6.44 mm vs. 7.47 mm). The results for suture displacement with each method are shown in Figure 5.

### Location analysis

**Figure 6:**
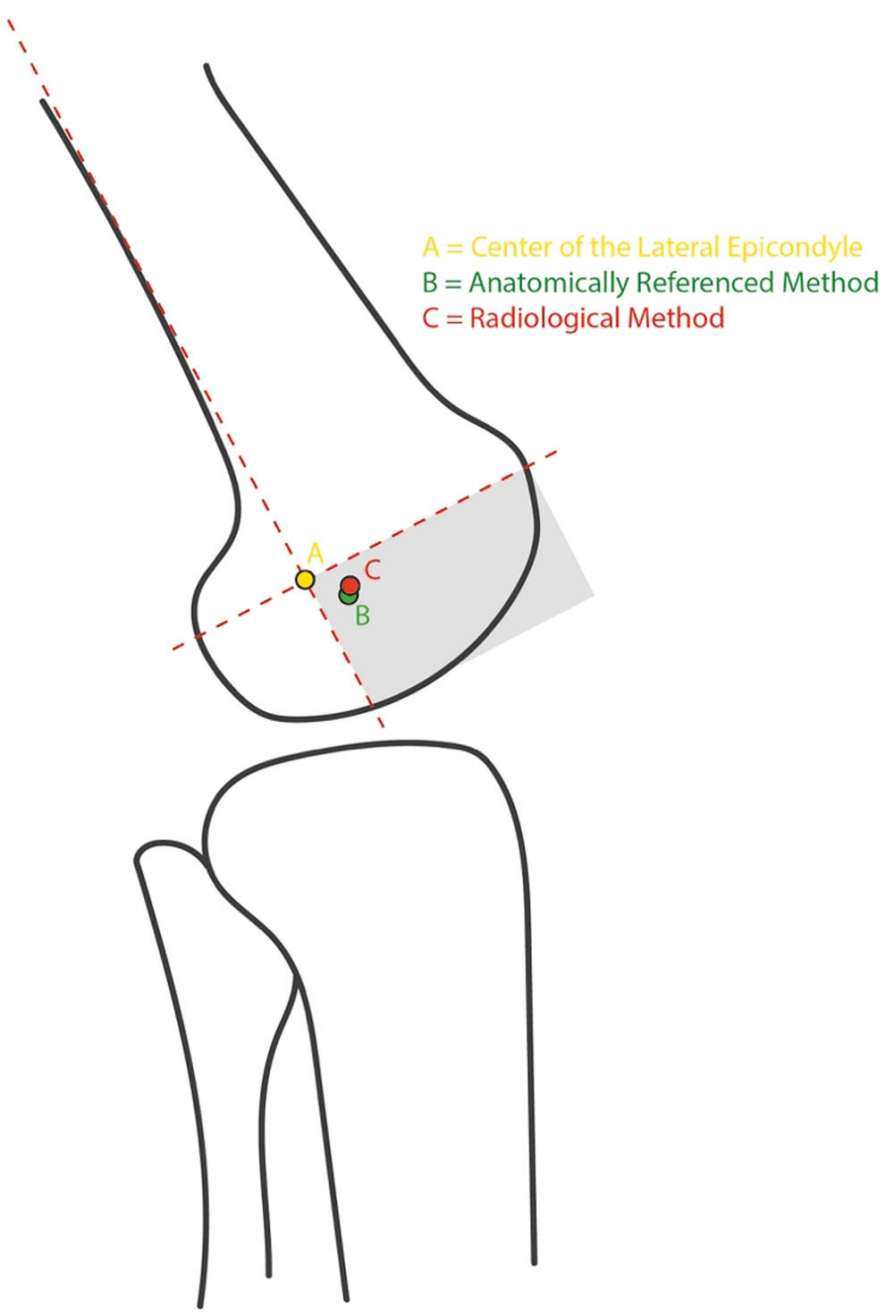
The median locations of the isometric graft insertion point (IGIP) identified using the anatomically referenced method (ARM, green point) and the radiological method (RM, red point), as well their relation to the centre of the lateral epicondyle (LE, yellow point). The intersection of the axes of the coordinate system is positioned at the centre of the LE. The anterior distal quadrant (grey‐shaded) contains both determined points.

When the straight lateral X‐rays of all the specimens were analysed, a location‐based relationship was observed between the position of the ARM, RM, and the centre of the LE. The median location of the ARM and RM was observed to be in the anterior distal quadrant of the xy‐coordinate system. The median location of the ARM was 6.5 mm (IQR 8.375 mm) distal and 3.95 mm (IQR 6.9 mm) anterior to the centre of the LE. The median location of the RM was 5.45 mm (IQR 3.5 mm) distal and 4.55 mm (IQR 5.75 mm) anterior to the centre of the LE. The median locations of the ARM and RM and their anatomical relation to the LE are shown in Figure 6.

## DISCUSSION

The main finding of this biomechanical study was that the IGIP determined using the ARM was significantly closer to the theoretical ideal IGIP than the one determined by the RM. In addition, the common location for the ARM was found to be 6.5 mm distal and 3.95 mm anterior to the centre of the LE.

Identifying the isometric point at the lateral femoral condyle is a challenging task, and methods of locating it are still a matter of controversy. A review of the literature shows that a maximum of only 2 mm of displacement has been declared acceptable [13]. However, the source of this finding appears to be speculative and without any solid evidence. Furthermore, hardly any comparative studies have managed to stay within the postulated 2 mm threshold. Sigward et al. used graphical analysis to demonstrate that even the optimal isometric point would show at least 1.3 mm of displacement [17]. The displacements measured in the present study are larger in comparison with those of other studies. However, the range of knee motion used in the reference studies was 0°–90° of knee flexion [12, 17], while in our study, suture displacement was measured from 0°−120° of flexion, which is the range of motion required for the most functional activities [5]. Additionally, importance of this distinction is also shown by Victor et al., who demonstrated that the lateral collateral ligament (LCL) follows almost isometric behaviour between 0° and 70° of flexion, but is subject to a length change from 70° to 120° of knee flexion [23]. Since the anterior limb in the MLT represents the function of the LCL which is known to undergo length changes at higher flexion grades, its insertion point should also be examined at higher flexion grades to accurately determine its optimal location [10, 11].

In the literature, anatomical and radiological methods for lateral knee reconstruction techniques have been compared, with contradictory results. In agreement with the findings of the present study, Leiter et al. showed that the anatomic method was more accurate for identifying isometry than the radiological method [12]. In their study, the authors evaluated the accuracy and reliability of the two methods, comparing the estimated isometric points in each method with the most isometric point. The present finding and the study by Leiter et al. contrast with the results reported by Stannard et al., who found that the radiological method was more accurate than the anatomical method for identifying the isometric point for lateral knee reconstruction with a fibular tunnel [18]. In our in vitro investigation, the anatomical approach was more accurate in finding the IGIP. However, in a clinical setting, the RM could prove to be an essential alternative, especially in cases of knee dislocation, as it can be reproducibly restored using intraoperative x‐ray imaging.

In the present study, the common location for the ARM and the RM were found to be 6.5 mm distal and 3.95 mm anterior, as well as 5.45 mm distal and 4.55 mm anterior to the centre of the LE, respectively. The two points are located close to each other, which shows that even small differences in distance can result in large differences in isometric behaviour. To the best of our knowledge, this is the first study that has reported on the location of the ARM and RM relative to the centre of the LE. It is important to recognise and extract the clinical relevance, since both, the ARM and the RM were found to be located in the anterior‐distal quadrant. Under normal and even more in challenging intraoperative conditions this information provides guidance were to begin to search for the IGIP.

Brinkman et al. documented the insertion geometry of the LCL and the popliteus tendon (PT), which are known to be the primary restraint structures for the posterolateral corner and are in contact with the femoral condyle [3, 15]. The authors found that the LCL inserts 4.6 mm posterior and 1.3 mm proximal to the LE, whereas the PT inserts 11 mm distally and slightly anterior to the LCL insertion point. La Prade et al. reported similar findings, noting that the insertion of the LCL was 3.1 mm posterior and 1.4 mm proximal to the LE, whereas the PT inserts at a mean of 18.5 mm anteriorly and distally to the femoral LCL attachment [8]. These findings demonstrate a close positional relationship between the anatomical insertion points of the LCL/PT and the IGIP for the modified Larson technique. Considering the complex anatomy and biomechanically synergistic behaviour of the LCL and PT [24], it seems unrealistic to reduce isometry to a single point on the lateral femoral condyle. Following the results of femoral insertion site studies [3, 8], the IGIP should be located somewhere between the insertion points of the LCL and the PT, slightly anteriorly and distally to the LE, as is reflected in the results of the present study.

Reporting on the anterior‐distal location of the IGIP in relation to the LE should help surgeons identify the IGIP more easily when performing MLT. The use of soft‐tissue landmarks could possibly be problematic, due to tissue distortions caused by multiligament knee injuries [8, 20]. The location of the ARM in relation to the LE thus appears to provide a reliable reference point, since in contrast to soft tissue, the LE is a prominent bony landmark that is not subject to disruption phenomena.

This study has some limitations. Given that this is a biomechanical study that provides a standardised in vitro evaluation for this geometrical research question, only time‐zero conditions are reported and results cannot be directly reproduced or fully compared to clinical studies. In the experimental setting, overview of the structures is obviously better due to the more extended dissection. Furthermore, the donors of the specimens used for the study were older than the typical patient population suffering from knee injuries. Another limitation of this study is the relatively small sample size, with specimens from eight donors; Although this is consistent with the sample sizes commonly reported in similar biomechanical studies [9, 12, 13, 14, 16, 21, 22], the results should be interpreted with care. In addition, limited number of specimens does not fully capture the variability in femoral geometry present in the general population. As a result, the location of the measured ideal insertion point might not fully cover the range of the general population.

In conclusion, the ARM was able to approximate the lateral femoral IGIP more closely than the RM. During surgery using the MLT, it may be recommended to start anteriorly and distally to the LE for femoral graft tunnel positioning.

## AUTHOR CONTRIBUTIONS

Christian Coppola, Maximilian Sigloch and Werner Schmoelz conceived and planned the experiments. Christian Coppola, Maximilian Sigloch and Werner Schmoelz carried out the experiments. Romed Hörmann, Christian Coppola and Maximilian Sigloch contributed to sample preparation. Christian Coppola, Maximilian Sigloch, Romed Hörmann, Werner Schmoelz and Raul Mayr contributed to the interpretation of the results. Christian Coppola, Werner Schmoelz and Raul Mayr took the lead in writing the manuscript. All authors provided critical feedback and helped shape the research, analysis and manuscript. All authors reviewed the final version of the manuscript.

## CONFLICT OF INTEREST STATEMENT

The authors declare no conflicts of interest.

## ETHICS STATEMENT

The tissue used for the experiments was harvested form bodies donated to the Department of Anatomy of the Medical University Innsbruck. The donors gave their written informed consent for the use of their body for teaching and research purposes during their lifetime. According to local laws an ethical statement is not required for tissue from bodies donated to the local Department of Anatomy.

## Data Availability

The data that support the findings of this study are available from the corresponding author upon reasonable request.
